# Pleth variability index or stroke volume optimization during open abdominal surgery: a randomized controlled trial

**DOI:** 10.1186/s12871-018-0579-4

**Published:** 2018-08-18

**Authors:** Hans Bahlmann, Robert G. Hahn, Lena Nilsson

**Affiliations:** 1 Department of Medical and Health Sciences, Linköping University, University Hospital, 58185 Linköping, Sweden; 2 Department of Anesthesiology and Intensive Care, Linköping University, University Hospital, 58185 Linköping, Sweden; 3 Department of Medical and Health Sciences, Linköping University and Research Unit, Södertälje Hospital, 152 86 Södertälje, Sweden

**Keywords:** Doppler ultrasonography, Fluid therapy, Laparotomy, Photoplethysmography, Stroke volume, Complications

## Abstract

**Background:**

The impact of Goal Directed Fluid Therapy (GDFT) based on the non-invasive Pleth Variability Index (PVI) on clinical outcome after abdominal surgery has only sparingly been explored. The purpose of this study was to compare the effect of intraoperative GDFT guided by PVI to a control group using esophageal Doppler on the incidence of complications and length of hospital stay after major abdominal surgery. We hypothesized that there would be no difference between the groups.

**Methods:**

This was a randomized controlled trial in a Swedish university hospital between November 2011 and January 2015; 150 patients scheduled for open abdominal surgery lasting 2 h or more were included. Exclusion criteria included hepatic resection or severe cardiac arrhythmia. The patients were randomized 1:1 to either the intervention group or the control group. The intervention group received intraoperative GDFT by administering fluid boluses of 3 ml/kg tetrastarch aiming at a PVI value below 10%, while GDFT in the control group aimed for optimization of stroke volume as assessed with esophageal Doppler. Blinded observers assessed complications until postoperative day 30 using pre-defined definitions, as well as length of hospital stay.

**Results:**

One hundred and-fifty patients were randomized and 146 patients were available for the final data analysis. Median duration of surgery was 3 h. A total of 64 complications occurred in the PVI group (*N* = 74) and 70 in the Doppler group (*N* = 72) (*p* = 0.93). Median (IQR) length of stay was 8.0 (8.0) days in the PVI group and 8.0 (9.5) in the Doppler group (*P* = 0.57).

**Conclusions:**

No difference in clinical outcome, as defined by number of postoperative complications, and length of hospital stay, was found when goal directed fluid therapy was applied using PVI as an alternative to esophageal Doppler. PVI appears to be an acceptable alternative to esophageal Doppler for goal directed fluid therapy during major open abdominal surgery.

**Trial registration:**

Clinicaltrials.gov NCT01458678. Date of first registration October 20, 2011.

**Electronic supplementary material:**

The online version of this article (10.1186/s12871-018-0579-4) contains supplementary material, which is available to authorized users.

## Background

The incidence of postoperative complications after major abdominal surgery is increased by both overly restricted and overly liberal fluid administration [1]. Goal directed fluid therapy (GDFT) aims to determine the optimal amount of fluid for an individual patient, and meta-analyses point out its clinical benefits, especially in patients not participating in an enhanced recovery program [2, 3]. Hemodynamic optimization has mostly been guided by stroke volume, commonly measured via esophageal Doppler, or by dynamic parameters such as stroke volume variation, pulse pressure variation or the pulse oximetric Pleth Variability Index (PVI), which all are based on cardiopulmonary interactions.

The latter techniques have been advocated because they are easier to apply and of similar clinical value as methods aiming at optimization of stroke volume [3] despite certain methodological issues, such as the influence of tidal volume, respiratory and heart rate ratio, spontaneous breathing, chest wall compliance, arrhythmia and abdominal pressure [4, 5]. The impact of PVI on clinical outcome in terms of postoperative complications or length of stay is sparingly described in randomized studies [6–8]. PVI guided bolus doses were compared either to a fluid regimen using bolus doses based on mean blood pressure [6, 7] or on Doppler measured stroke volume changes [8]. Although no difference on the incidence of postoperative complications or length of stay was reported, the studies are small and two of them restricted to colonic surgery. We found it of interest to further evaluate the effect of PVI on clinical outcome in abdominal surgery.

We designed a randomized controlled trial comparing patients treated with fluid optimization guided by PVI with control patients who received stroke volume optimization guided by esophageal Doppler.

This is a planned secondary analysis of a study primarily designed to analyze differences in intraoperative fluid use. Primary outcome measures were the amount of colloid used for optimization of fluid status and the concordance between different methods for assessing preoperative dehydration. These results have been published elsewhere [9, 10] and included the first half of the study cohort (75 patients). The present article focuses on clinical outcome in the whole cohort of 150 patients, as quantified by number of postoperative complications up to 30 days after surgery and length of hospital stay, both of which were designated as secondary outcome measures in the study protocol.

We hypothesized that there would be no difference between the groups regarding these parameters.

## Methods

Ethical approval was obtained from the Regional Ethical Review Board in Linköping in March 2011 (2011/101–31), as was written informed consent from all included patients. The study was prospectively registered at clinicaltrials.gov (NCT 01458678).

This single-blind randomized study was performed at University Hospital Linköping, a 600-bed tertiary care facility in Sweden. As previously described [9], adult patients, ASA class 1–3, planned for elective open abdominal surgery with an expected duration of at least 2 h were screened for inclusion. Exclusion criteria included hepatic resection, severe forms of cardiac arrhythmia, planned central hemodynamic monitoring for cardiac reasons, enrollment in another interventional study or unavailability of research staff.

### General management

Patients were recruited from different departments (gynecology, surgery and urology) and therefore preoperative management with regard to fluids and antibiotics followed departmental routines, which could include an explicit enhanced recovery program. After siting a thoracic epidural catheter (when indicated), general anesthesia was induced using fentanyl and propofol or thiopental and maintained using sevoflurane and iterated doses of fentanyl. Intubation was facilitated with rocuronium or succinylcholine. Epidural analgesia was started before the surgery commenced using a continuous infusion of a mixture of bupivacaine, fentanyl and epinephrine supplemented by bolus doses if indicated. All patients were ventilated using a Volume Control mode with a standardized tidal volume of 7 ml kg^− 1^ ideal body weight [11].

A maximum of 500 ml of tetrastarch, either Venofundin (B Braun Medical AB, Danderyd, Sweden) or Volulyte (Fresenius Kabi AB, Uppsala, Sweden), could be infused during the epidural catheter placement and the subsequent induction of anesthesia. Both groups received a baseline infusion of 2 ml kg^− 1^ h^− 1^ (actual body weight) of a 2.5% buffered dextrose solution containing 70 mmol l^− 1^ sodium, 45 mmol l^− 1^ chloride, and 25 mmol l^− 1^ acetate (Glukos Braun 25 mg ml^− 1^ with buffer (B Braun Medical AB, Danderyd, Sweden) during surgery. Correction for additional crystalloid fluids given (e.g. antibiotics) was not made. Up to 1000 ml of Ringer’s acetate could be infused during surgery to compensate for preoperative dehydration or increased non-hemorrhagic intraoperative fluid loss. Bleeding was replaced 1:1 with a colloid (tetrastarch, albumin 5%, plasma, packed red blood cells or platelets). Infusions with vasoconstrictors (norepinephrine or phenylephrine) and/or inotropes (dobutamine) were used at the discretion of the responsible anesthetist.

### Study groups

Patients were recruited and randomized 1:1 by HB or LN, using opaque envelopes prepared by a research nurse from a computerized randomization procedure [12], to receive intraoperative fluid optimization guided by either the pulse oximetric PVI or esophageal Doppler. The PVI was monitored using a Radical-7 Pulse CO-oximeter (Masimo Corporation, Irvine, California, USA) with PVI software (Version SET V7.8.0.1), a re-usable sensor (R2-25r) and a disposable adhesive (R2-25a). The sensor was placed on the middle or index finger of either hand and was covered to avoid room light interference. The placement of a blood pressure cuff on the same arm was avoided.

The esophageal Doppler measurements were performed using a CardioQ apparatus (Deltex Medical, Chicester, United Kingdom) equipped with a DP12 probe, and the signal was averaged over 20 cardiac cycles. In both groups, fluid optimization was initiated after induction of general anesthesia and undertaken by infusing 3 ml kg^− 1^ actual body weight (up to 250 ml) of tetrastarch intravenously during 3–5 min, using a 50 ml syringe. As described previously [9], during the first half of the study (patients 1–75), in which the concordance between the methods was studied, all patients were monitored with both PVI and esophageal Doppler. Only the allocated monitor was visible to the responsible anesthetist. For the second part (patients 76–150) only the allocated monitor was used.

### Fluid bolus algorithms

In the PVI group, a fluid bolus was given if PVI ≥ 10%. The cut-off value of 10% was chosen based on a previous report [13]. If the PVI 5 min after the fluid bolus fell below 10%, no more fluid was given. If the PVI 5 min after the fluid bolus was still ≥10% but had decreased, a repeat fluid bolus was given. Fluid boluses were repeated until the PVI fell below 10% or did not decrease at all. During surgery, additional optimizations were undertaken in the same way whenever the PVI increased to ≥10%. In order to have at least one fluid bolus available in each patient for analyzing the performance of PVI compared to Doppler during an optimization and in analogy with the Doppler group [9], a fluid bolus was given to all patients in the PVI group after induction of anesthesia, irrespective of the PVI value.

In the Doppler group, volume optimization was guided by the stroke volume changes in accordance with published protocols [14, 15]. Following an initial Doppler measurement, a fluid bolus was given and a new Doppler measurement was performed 5 min later. Fluid boluses were repeated until the stroke volume no longer increased by 10%. The 10% cut-off value is commonly used in studies involving the esophageal Doppler, and is based on measurement characteristics of the device [16]. During surgery, additional optimizations were undertaken in the same way whenever the stroke volume had decreased by ≥10%.

### Clinical outcome

Two blinded observers retrospectively documented complications during the first 30 days after surgery using a pre-specified list of complications adapted from Brandstrup et al. [17] (see Additional file 1). A telephone call from the research nurse after postoperative day 30 supplemented the information from the electronic patient chart. If patients received care at another hospital during the 30 day period, copies of relevant reports were requested. Each observer independently scored complications; differences were resolved by discussion.

Length of hospital stay was defined as the number of calendar days spent in hospital, from the day of primary surgery until the 30th postoperative day. In case of in-hospital death within the observation period, a length of stay of 31 days was assumed.

### Statistical analysis

In order to calculate a sample size that would be sufficient also for the secondary endpoint postoperative complications, we used combined data from the intervention arms of five previous studies on GDFT during abdominal surgery [18–22]. An expected total of 55 complications per 100 patients with a standard deviation of 19 was calculated. With a power of 90% and a significance level of 5%, 66 patients would need to be included in each group to be able to demonstrate an absolute difference of 10% in postoperative complications [23]. Allowing for dropouts it was decided to include 150 patients in the study. Analysis was done on an intention to treat basis. Between groups differences were analyzed using Student T-test, Mann-Whitney U-test, Fisher’s Exact test and chi-square test as appropriate using Statistica, versions 12 and 13 (Dell Incorporated, Tulsa, Oklahoma, USA). A *p*-value < 0.05 was considered significant.

## Results

**Fig. 1:**
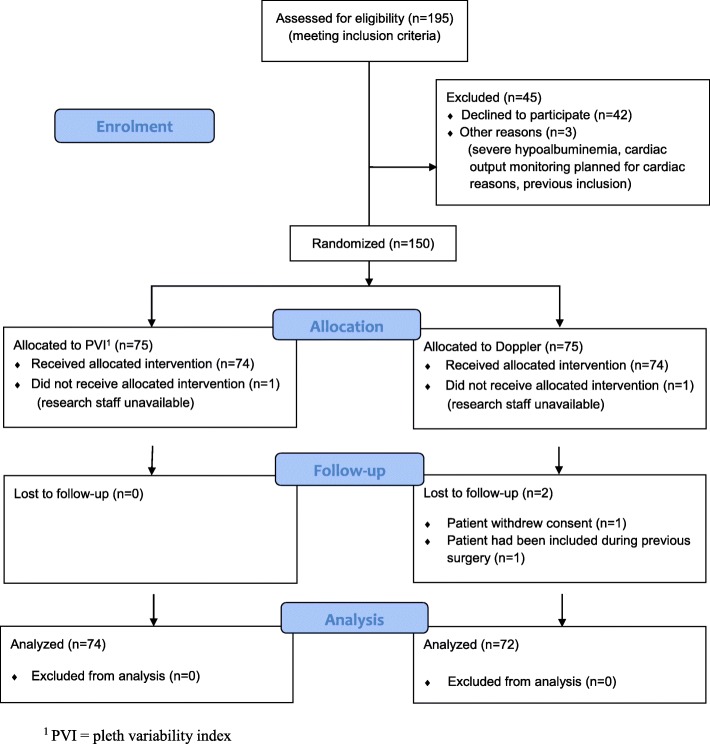
CONSORT 2010 Flow Diagram

Patients were recruited between November 14, 2011 and December 8, 2014, and the follow up of the last patient ended on January 8, 2015. A CONSORT flow diagram is presented in Fig. 1. After exclusion of four patients 146 were available for analysis.

Patient and surgical characteristics are presented in Table 1. Median duration of surgery was 3 h in both groups, with some procedures lasting more than 12 h. Types of surgery are specified in Additional file 2.

**Table 1:** Patient characteristics and perioperative data

	PVI (*n* = 74)	Doppler (*n* = 72)
Age, mean (SD), years	63 (12)	61 (14)
Female, n (%)	40 (54)	47 (65)
Body mass index, mean (SD), kg m^− 2^	26.0 (4.1)	26.7 (6.3)
ASA class		
ASA 1, n (%)	22 (30)	27 (38)
ASA 2, n (%)	42 (57)	37 (51)
ASA 3, n (%)	10 (13)	8 (11)
Concomitant morbidity		
Smoker, n (%)	14 (19)	8 (11)
Cardiovascular disease, n (%)	31 (42)	22 (31)
Neurological disease, n (%)	4 (5)	2 (3)
Diabetes, n (%)	11 (15)	8 (11)
Type of surgery		
Gynecology, n (%)	23 (31)	19 (26)
Upper gastrointestinal, n (%)	18 (24)	26 (36)
Lower gastrointestinal, n (%)	22 (30)	21 (29)
Urology, n (%)	11 (15)	6 (9)
Enhanced recovery program, n (%)	7 (9)	8 (11)
Duration of surgery, median {IQR}, h	2.9 {2.2}	3.0 {2.2}
Duration of anesthesia, median {IQR}, h	4.1 {2.3}	3.9 {2.3}
Epidural, n (%)	66 (89)	67 (93)
Lowest temperature during surgery, mean (SD), °C	35.7 (0.5)	35.7 (0.6)
Temperature end of surgery, mean (SD), °C	36.4 (0.6)	36.4 (0.6)

In two patients in the PVI group (one with increasing lactic acidosis and one with continuing hemodynamic instability), in which the PVI indicated no need for further fluids, Doppler data were requested by the responsible anesthetist. These data confirmed an adequate fluid status, and the Doppler data were blinded for the rest of the procedure. These patients remained in the PVI group for the intention to treat analysis.

There were no differences between the groups in the amounts of colloids used during the optimizations, or in any other intraoperative fluid parameters (Table 2), with the exception of phenylephrine slightly more often being used in the PVI group (*P* = 0.04). Mean increase in body weight 1 day after surgery was 1.3 kg (SD 2.4) in the PVI group and 1.3 kg (SD 1.7) in the Doppler group (*P* = 0.97).

**Table 2:** Intraoperative fluid data

	PVI (*n* = 74)	Doppler (*n* = 72)	*P*
Crystalloid fluid, mean (SD), ml	1360 (749)	1240 (662)	0.31
Total colloid fluid, mean (SD), ml	1464 (1000)	1412 (1259)	0.92
Colloid during induction, mean (SD), ml	173 (145)	154 (137)	0.40
Colloid used during optimizations, mean (SD), ml	675 (434)	665 (462)	0.89
Synthetic colloid fluid, mean (SD), ml	1159 (507)	1141 (532)	0.84
Albumin 5%, n (range, ml)	14 (120–1250)	8 (170–500)	0.25
Albumin 20%, n (range, ml)	8 (100–200)	6 (45–100)	0.78
Red blood cells, n (range, ml)	10 (280–1389)	8 (265–2310)	0.80
Plasma, n (range, ml)	6 (776–2734)	5 (265–3300)	1.00
Thrombocytes, n (range, ml)	0	2 (230–250)	0.24
Phenylephrine, n (range, μg)	51 (360–3928)	37 (240–6640)	**0.04**
Norepinephrine, n (range, μg)	29 (52–2180)	30 (11–2726)	0.76
Dobutamine, n (range, mg)	20 (2–110)	20 (1–97)	0.92
Blood loss, median {IQR}, ml	250 {500}	225 {487}	0.41
Urine, median {IQR}, ml	300 {326}	225 {310}	0.22

Statistical differences were analyzed using Student T-test, Mann-Whitney U-test, chi-square test or Fisher’s Exact test as appropriate. Significant results in bold

### Postoperative complications and length of hospital stay

There was no mortality during the study period. There were in total 64 complications in the PVI group (*n* = 74) and 70 in the Doppler group (*n* = 72) (*P* = 0.93), corresponding to 38 (51%) patients in the PVI group and 35 (49%) patients in the Doppler group (*P* = 0.74) having had at least one complication (Table 3). Eleven complications necessitated surgical intervention requiring general anesthesia (three in the PVI group and eight in the Doppler group (*P* = 0.55)) and in each group two complications required admission to the intensive care unit. Median length of hospital stay was 8.0 days (IQR 8.0) in the PVI group and 8.0 days (IQR 9.5) in the Doppler group (*P* = 0.57*).*

**Table 3:** Postoperative complications within 30 days after surgery

	PVI (*n* = 74)	Doppler (*n* = 72)	
Major			
Anastomotic insufficiency	3	1	
Lymphatic leakage	0	0	
Bleeding	0	1	
Sepsis	0	1	
Wound dehiscence	0	5	
Intestinal obstruction	0	1	
Stroke	0	0	
Pulmonary embolism	0	0	
Deep vein thrombosis	1	1	
Pulmonary edema/ respiratory insufficiency/ pneumonia	1	2	
Pleural effusion	0	5	
Myocardial infarction	1	0	
Arrhythmia	1	2	
Cardiac arrest	0	0	
Renal dysfunction	13	10	
Liver dysfunction	0	0	
Total	20	29	
Minor			
Superficial wound infection or dehiscence	6	4	
Infection	10	11	
Paralytic ileus	1	0	
Upper GI bleeding	0	1	
Pulmonary congestion	5	0	
Angina pectoris	1	1	
Hypotension	2	6	
Delirium	1	1	
Coagulopathy	4	3	
Severe postoperative nausea and vomiting	8	9	
Urinary retention	6	5	
Total	44	41	
Total number of complications	64	70	(*P* = 0.93)
Number (%) of patients with complications	38 (51)	35 (49)	(*P* = 0.74)
Mean number of complications in patients with complications	1.7	2.0	(*P* = 0.28)

Statistical differences were analyzed using Mann-Whitney U-test or chi-square test as appropriate

## Discussion

There were no differences in the number of postoperative complications or length of hospital stay when GDFT was guided by PVI instead of esophageal Doppler during major open abdominal surgical procedures.

Previous studies show that both length of stay and incidence of complications were reduced when PVI was used as a part of a colorectal surgery multimodal enhanced recovery protocol in 109 patients, compared to historical controls [24]. The use of PVI also decreased perioperative lactate levels compared to controls [6, 25]. Warnakulasuriya et al. [8] reported no difference in short term outcome when PVI was compared to esophageal Doppler in 40 patients. Based on these reports and our findings, a clinician wishing to pursue GDFT can choose PVI over esophageal Doppler in the large majority of patients undergoing major open abdominal surgery. PVI, in contrast to esophageal Doppler, is not sensitive to interference from diathermy and does not require frequent access to the patient’s head for probe repositioning. Also, PVI can be measured without single-use equipment avoiding the cost for single-use esophageal probes (USD 130 in our setting). However, as illustrated by the request for Doppler data in two PVI patients, the clinician might still want to have access to a reliable method for measuring intraoperative cardiac output to increase the amount of hemodynamic information during unusually complex situations or in cases of known vascular and/or myocardial dysfunction. Data on cardiac output and thus contractility, systemic vascular resistance and oxygen delivery were available in the Doppler group but not incorporated in the treatment protocol, and decisions about inotropic and/or vasoactive support in both groups were left to the clinical judgement of the responsible anesthetist. Despite the more extensive hemodynamic information in the Doppler group, beside more use of the vasopressor phenylephrine in the PVI group, the amount of inotropic support did not differ between the treatment groups.

Other non-invasive methods for dynamic monitoring are available. One meta-analysis reported the offline assessment of respiratory variation in pulse oximetry plethysmographic waveform amplitude (∆POP) for predicting fluid responsiveness to be comparable to that of PVI (AUC 0.89 vs 0.95), but this technique is as yet not available in real time [26]. Also, pulse pressure variation and stroke volume variation can now be obtained non-invasively using the Nexfin/Clearsight™ or the CNAP™ systems, but their capacity for predicting fluid responsiveness differs between studies [27–30]. The same applies to measurements of the variability of the diameter of the caval vein or other veins during positive pressure ventilation [31]. Contrary to PVI, the effect on clinical outcome of all these techniques has to the best of our knowledge not been studied.

PVI is a dynamic indicator of fluid responsiveness based on cardiopulmonary interaction while stroke volume optimization is not. As both methods are claimed to be of benefit for fluid management, we argue that these methods, though differing in concept, can be compared with each other in a randomized trial focusing on clinical outcome [3]. Maintaining a patient below a certain PVI value might appear to be less aggressive then aiming for the plateau of the Starling curve, but this depends on the selected cut-off values. Probably, a higher cut off value for PVI with the same settings (such as tidal volume) would have resulted in less fluid administration and vice versa. We have previously shown that a PVI value of 10.5% was optimal in this material [9].

### Limitations

Limitations of this study include the absence of a control group without either PVI or Doppler to guide volume optimization. Early reports on stroke volume optimization using the esophageal Doppler were favorable [32]. Since then the technique has been questioned, partly because of negative studies on GDFT involving esophageal Doppler [33] and other devices [34, 35], and partly because of questions raised about the Doppler method itself [36]. Based on our results, it is not possible to tell whether PVI and Doppler both result in similar improvements in outcome, or whether neither method improves outcome when compared to treatment without GDFT. Such a control group was reflected upon at the time of designing the study (2011), but deemed unethical since, based on the evidence available at that time, we felt there was no equipoise about the benefits of GDFT. Equipoise about the use of GDFT is supported by our findings previously reported about the limited ability of both PVI and Doppler to predict fluid responsiveness [9]. Also protocol compliance, in terms of time during surgery with achieved PVI and SV goals respectively, was not recorded.

The amount of fluid used during the optimizations represents a part of the total fluid given, because of the per protocol complementary colloid fluid administration during the induction of anesthesia and for bleeding, and crystalloid fluid for maintenance and for the correction of preoperative dehydration. This increased the possibility that both groups would receive similar amounts of fluid; however, we did not find a difference between the groups in the amount of colloids used specifically for the optimizations.

We selected a difference of 10% in number of complications at 30 days to determine required sample size. Therefore the study was not powered to detect smaller albeit still significant differences in postoperative outcome. Also, it could be discussed whether a non-inferiority approach would have been more adequate. This would entail a larger required sample size.

The studies which were used to determine sample size scored complications in different ways and reported incidences of complications which are higher than can be expected in current practice using improved surgical and anesthetic techniques. The findings of this study cannot be applied to laparoscopic surgery. The majority of patients, although undergoing major surgery, were classified as ASA 1 or 2. The potential of GDFT to improve postoperative outcome is more pronounced in patients at higher risk of complications [37], and this could have influenced the possibility to find differences in outcome between the methods. Also, since intraoperative blood pressure is correlated to postoperative outcome, a target MAP should have been specified in the protocol.

Since the reliability of dynamic parameters including PVI increases with increasing tidal volume, a tidal volume of 8 ml kg^− 1^ (ideal or actual body weight) or more is often recommended [38]. However, as large tidal volumes are probably detrimental in surgical patients [39] we, in line with earlier reports evaluating PVI, chose a tidal volume of 7 ml kg^− 1^ ideal body weight [6, 7, 13]. Whether or not outcome is improved by using larger tidal volumes in surgical patients monitored with dynamic parameters is unknown.

## Conclusions

We found no differences regarding number of postoperative complications or length of hospital stay between using PVI or esophageal Doppler for goal directed fluid therapy. PVI appears to be an acceptable alternative to esophageal Doppler for goal directed fluid therapy during major open abdominal surgery.

## Additional files

Additional file 1: Definition of complications. Description of the criteria applied when scoring complications. (DOCX 18 kb)

Additional file 2: Types of surgery. List of surgical procedures performed during the study. (DOCX 16 kb)

## Abbreviations

ASA: American Society of Anesthesiologists
CONSORT: Consolidated standards of reporting trials
ECG: Electrocardiogram
GDFT: Goal-directed fluid therapy
IQR: Interquartile range
LBBB: Left bundle branch block
MAP: Mean arterial pressure
PaCO_2_: Partial pressure of carbon dioxide in arterial blood
PaO2/FiO2: Ratio of partial pressure of oxygen in arterial blood divided by fraction of inspired oxygen
PT (INR): Prothrombin time (international normalized ratio)
PVI: Pleth variability Index
SD: Standard deviation
