# Supplementary material for: Pleth variability index or stroke volume optimization during open abdominal surgery: a randomized controlled trial
Source: BMC Anesthesiol. 2018 Aug 18;18:115. doi: 10.1186/s12871-018-0579-4 (PMC6098821; doi:10.1186/s12871-018-0579-4)
Supplement: Supplementary file 1 — Definition of complications. Description of the criteria applied when scoring complications. (DOCX 18 kb) [file 12871_2018_579_MOESM1_ESM.docx]

Additional File 1. Definition of complications.

| **Major complications** | Requirement |
| --- | --- |
| Anastomotic insufficiency | Requiring operation |
| Lymphatic leakage | Requiring operation |
| Bleeding | Requiring operation |
| Sepsis | Severe sepsis or septic shock  Three or more of the following signs:   - Temperature > 38 or < 36 °C - Heart rate > 90 min^-1^ - Respiratory rate > 20 min^-1^ or P_a_CO_2_ < 4.3 kPa or ventilator treatment - White blood cells > 12.000 µl^-1^ or < 4.000 µl^-1^ or > 10% immature cells   At least two organ dysfunctions > 24 h   - Circulatory: Systolic Blood Pressure < 90 mmHg or Mean Arterial Pressure (MAP) < 70 mmHg for at least one hour, despite adequate fluid resuscitation, or stable hemodynamics (systolic Blood Pressure > 90 mmHg and MAP > 70 mmHg) using catecholamines. - Respiratory: P_a_O_2_/F_i_O_2_ < 33.3 kPa or P_a_O_2_/F_i_O_2_ < 27 kPa if primary pulmonary condition - Renal: Diuresis < 0.5 ml kg^-1^ h^-1^, or > 50% increase in serum creatinine above baseline, or dialysis - Coagulation: Platelet count < 80.000 µl^-1^ or > 50% decrease within 3 days - Metabolic: pH < 7.3 or Base Excess > - 5 combined with serum lactate > 1.5 times normal value |
| Wound dehiscence | Suture of the fascia |
| Intestinal obstruction | Requiring dilatation or operation |
| Stroke | Clinical symptoms and findings on computed tomography |
| Pulmonary embolism | Sudden death or findings on computed tomography |
| Deep vein thrombosis | Requiring treatment, without pulmonary embolism |
| Pulmonary edema/ respiratory insufficiency/ pneumonia | Ventilator treatment or Continuous Positive Airway Pressure / Non-Invasive Ventilation not exclusively for prophylaxis of atelectasis |
| Pleural effusion | Drainage |
| Myocardial infarction | 1. Increase in serum levels of high-sensitivity Troponin T above 15 ng l^-1^, and an increasing or decreasing trend in these serum levels based on at least two samples with at least a 6-hour interval, and at least one of the following:  a. Typical symptoms: Typical chest pain for at least 15 minutes or pulmonary edema without any other cause.  b. ECG changes: Appearance of pathological Q-waves (duration > 0.03 sec and > 25% of R-wave amplitude) in at least 2 leads, or ischemic ST-changes or appearance of left bundle branch block (LBBB).  c. Imaging studies showing recent loss of viable myocardium or appearance of regional wall motion abnormality,  or  2. Typical symptoms and ST increase/new LBBB/new thrombus as seen on coronary angiography and no possibility for further work-up because of sudden death. |
| Arrhythmia | Requiring medical treatment or electro-conversion |
| Cardiac arrest | Cardiopulmonary resuscitation |
| Renal dysfunction | Diuresis < 500 ml 24 h^-1^, > 30% increase in serum creatinine compared to baseline |
| Liver dysfunction | Serum bilirubin > 100 µmol l^-1^ and prothrombin time (international normalized ratio) (PT (INR)) > 1.6 |
|  |  |
| **Minor Complications** |  |
| Superficial wound infection or dehiscence | Local inflammatory signs and specific antibiotic treatment |
| Infection | Fever and new antibiotic treatment not qualifying as sepsis |
| Paralytic ileus | > 7 days without flatus |
| Upper GI bleeding | Clinical and/or endoscopic signs and specific treatment |
| Pulmonary congestion | Dyspnea and auscultatory findings compatible with pulmonary congestion, and pharmacological treatment |
| Angina pectoris | Pharmacological treatment |
| Hypotension | Requiring pharmacological treatment > 12 h |
| Delirium | Requiring intervention |
| Coagulopathy | Platelet count < 100.000 µl^-1^, PT (INR) > 1.6, activated partial thromboplastin time. > 50 s |
| Severe postoperative nausea and vomiting | Nausea and/or vomiting limiting mobilization, and/or requiring iv fluids, and/or causing prolonged length of stay |
| Urinary retention | Urinary retention requiring catheter (excluding catheter as required by local protocol) |
