# Supplementary material for: Pleth variability index or stroke volume optimization during open abdominal surgery: a randomized controlled trial
Source: BMC Anesthesiol. 2018 Aug 18;18:115. doi: 10.1186/s12871-018-0579-4 (PMC6098821; doi:10.1186/s12871-018-0579-4)
Supplement: Supplementary file 2 — Types of surgery. List of surgical procedures performed during the study. (DOCX 16 kb) [file 12871_2018_579_MOESM2_ESM.docx]

Additional File 2. Types of surgery

|  | PVI (n = 74) | Doppler (n = 72) |
| --- | --- | --- |
| **Gynecological procedures** |  |  |
| Hysterectomy with salpingo-oophorectomy | 17 | 15 |
| Wertheim procedure | 3 | 1 |
| Salpingo-oophorectomy | 2 | 1 |
| Interval surgery | 1 | 0 |
| Lymph node dissection | 0 | 1 |
| Excision of vesicovaginal fistula | 0 | 1 |
|  |  |  |
| **Upper gastrointestinal procedures** |  |  |
| Pancreatectomy including Whipple procedure | 10 | 12 |
| Gastrectomy | 3 | 4 |
| Gastric resection | 3 | 2 |
| Excision of retroperitoneal tumour | 1 | 3 |
| Small bowel resection | 0 | 2 |
| Repair of diaphragmatic hernia | 1 | 0 |
| Excision of sarcoma | 0 | 1 |
| Cholecystectomy | 0 | 1 |
| Hepaticojejunostomy | 0 | 1 |
|  |  |  |
| **Lower gastrointestinal procedures** |  |  |
| Sigmoid resection | 3 | 4 |
| Proctectomy with or without pelvic reservoir | 2 | 2 |
| Small bowel resection | 1 | 3 |
| Reversal of colostomy or ileostomy | 2 | 2 |
| Hemicolectomy | 2 | 2 |
| Total mesorectal excision | 1 | 2 |
| Lower anterior resection | 1 | 2 |
| Excision of enterocutaneous fistula | 3 | 0 |
| Total colectomy | 2 | 0 |
| Rectum resection | 1 | 1 |
| Revision of pelvic reservoir | 1 | 1 |
| Creation of pelvic reservoir | 1 | 1 |
| Revision of stomy | 1 | 0 |
| Repair of incisional hernia | 0 | 1 |
| Rectosigmoid resection | 1 | 0 |
|  |  |  |
| **Urological procedures** |  |  |
| Renal resection | 3 | 4 |
| Nephrectomy | 4 | 0 |
| Cystectomy with reservoir | 2 | 1 |
| Ureteroenterostomy with reservoir | 0 | 1 |
| Pelvic lymph node dissection | 1 | 0 |
| Revision of pelvic reservoir | 1 | 0 |
